# The Site of Origin of Medulloblastoma: Surgical Observations Correlated to Molecular Groups

**DOI:** 10.3390/cancers15194877

**Published:** 2023-10-07

**Authors:** Olga Ciobanu-Caraus, Thomas Czech, Andreas Peyrl, Christine Haberler, Gregor Kasprian, Julia Furtner, Marcel Kool, Martin Sill, Josa M. Frischer, Anna Cho, Irene Slavc, Karl Rössler, Johannes Gojo, Christian Dorfer

**Affiliations:** 1 Department of Neurosurgery, Medical University of Vienna, 1090 Vienna, Austriathomas.czech@meduniwien.ac.at (T.C.); anna.cho@meduniwien.ac.at (A.C.);; 2 Department of Pediatrics and Adolescent Medicine, Medical University of Vienna, 1090 Vienna, Austriairene.slavc@meduniwien.ac.at (I.S.);; 3 Comprehensive Center for Pediatrics and Comprehensive Cancer Center, Medical University of Vienna, 1090 Vienna, Austria; 4 Division of Neuropathology and Neurochemistry, Department of Neurology, Medical University of Vienna, 1090 Vienna, Austria; 5 Department of Radiology, Medical University of Vienna, 1090 Vienna, Austria; gregor.kasprian@meduniwien.ac.at (G.K.); julia.furtner-srajer@meduniwien.ac.at (J.F.); 6 Hopp Children’s Cancer Center (KiTZ), 69120 Heidelberg, Germany; 7 Division of Pediatric Neurooncology, German Cancer Research Center (DKFZ) and German Cancer Consortium (DKTK), 69120 Heidelberg, Germany; 8 Princess Máxima Center for Pediatric Oncology, 3584 Utrecht, The Netherlands

**Keywords:** MB, origin, molecular group, neurosurgery

## Abstract

**Simple Summary:**

Medulloblastoma is one of the most common malignant brain tumor types and comprises the molecular groups WNT-MB, SHH-MB TP53-wildtype, SHH-MB TP53-mutant and non-WNT/non-SHH MB. Developmental gene expression data suggest that these molecular groups arise from different sites of origin in the developing brain. Therefore, we hypothesized that intraoperative observation could enable the neurosurgeon to predict the molecular group during surgery and opt for a less aggressive resection in molecular groups with favorable prognosis to preserve neurological function. We reviewed the intraoperative reports, surgical videos and information on the molecular group of 58 patients who were operated at our institution from 1996 to 2020. Our data demonstrate that the correct prediction of the molecular group based on the intraoperatively observed site of origin was limited. Based on our results, we conclude that maximal safe resection should remain the aim of surgery irrespective of the observed site of origin.

**Abstract:**

Developmental gene expression data from medulloblastoma (MB) suggest that WNT-MB originates from the region of the embryonic lower rhombic lip (LRL), whereas SHH-MB and non-WNT/non-SHH MB arise from cerebellar precursor matrix regions. This study aimed to analyze detailed intraoperative data with regard to the site of origin (STO) and compare these findings with the hypothesized regions of origin associated with the molecular group. A review of the institutional database identified 58 out of 72 pediatric patients who were operated for an MB at our department between 1996 and 2020 that had a detailed operative report and a surgical video as well as clinical and genetic classification data available for analysis. The STO was assessed based on intraoperative findings. Using the intraoperatively defined STO, “correct” prediction of molecular groups was feasible in 20% of WNT-MB, 60% of SHH-MB and 71% of non-WNT/non-SHH MB. The positive predictive values of the neurosurgical inspection to detect the molecular group were 0.21 (95% CI 0.08–0.48) for WNT-MB, 0.86 (95% CI 0.49–0.97) for SHH-MB and 0.73 (95% CI 0.57–0.85) for non-WNT/non-SHH MB. The present study demonstrated a limited predictive value of the intraoperatively observed STO for the prediction of the molecular group of MB.

## 1. Introduction

Medulloblastoma (MB) is one of the most common malignant pediatric brain tumor types [1]. According to the current WHO classification of CNS tumors (2021), MB comprises four major molecular groups: MB, WNT-activated (WNT-MB); MB, SHH-activated (SHH-MB) TP53-wildtype; MB, SHH-activated TP53-mutant; and MB, non-WNT/non-SHH. Additionally, these four major groups have been shown to encompass further subgroups [2,3]. This molecularly defined grouping carries prognostic and potentially predictive value.

About a decade ago, developmental gene expression data first revealed that the molecular groups originate from distinct cell lineages in the developing brain [4]. According to this recently strengthened concept of group-specific cellular origins, WNT-MB presumably arises from the lower rhombic lip (LRL) of the embryonal brainstem, SHH-MB mainly from the external granule cell layer of the cerebellar hemispheres and non-SHH/non-WNT MB from the upper rhombic lip within the inferior cerebellar vermis [5].

To date, the standard of care for MB in children consists of gross total resection, followed by radiotherapy and chemotherapy in children over the age of three years [6,7]. During surgery, most neurosurgeons generally tend to aim for gross total resection whenever possible, although there is insufficient definitive evidence that maximizing the extent of resection is associated with improved progression-free survival and overall survival [8]. As MB is inherently situated in an area that harbors vital neurological functions, neurosurgeons are often confronted with the decision to either attempt gross total resection or preserve neurological function with the risk of impacting the patient outcome [9]. Intraoperatively, the knowledge of the molecular group would add valuable information to optimize and tailor the neurosurgical strategy to the prognostic profile of the patient. While several studies attempted to analyze imaging characteristics of the molecular groups [10,11,12,13,14,15,16,17], growth characteristics of MB groups have rarely been described from a neurosurgical perspective.

Based on the assumption that the molecular groups exclusively arise from distinct sites of origin (STOs) [4,18], we hypothesized that intraoperative observation could enable the neurosurgeon to predict the molecular group during surgery and thus allow a risk-stratified approach. Given the fact that pediatric WNT-MB is prognostically superior in comparison with all other molecular groups, it would be arguably justifiable to opt for a subtotal resection in order to preserve neurological function [19,20,21].

Therefore, this study aimed to identify the STO from a neurosurgical perspective and to correlate it to the molecular groups.

## 2. Materials and Methods

### 2.1. Study Population

One hundred and thirty-five pediatric (aged under 18 years at time of diagnosis) patients with a histologically confirmed MB who were referred to our Department of Pediatrics and Adolescent Medicine between 1996 and 2020 were identified from an institutional database. Of these, 72 patients were operated at our Department of Neurosurgery. Inclusion criteria for this study were the availability of surgical notes and of surgical video documentation allowing for an unequivocal definition of the tumor growth pattern. Fifty-eight patients could be included based on these criteria.

Patient demographics, presence of metastases at diagnosis, treatment modalities (number of surgeries, radiotherapy, chemotherapy), tumor size, pre- and postoperative symptoms and patient outcome were extracted from the medical charts. The extent of resection was evaluated according to interdisciplinary tumor board reviews, operative reports and MRI imaging performed 72 h postoperatively and classified as gross total/near-total resection (GTR/NTR) or subtotal resection (STR, residual tumor > 1.5 cm^2^). Patients with disseminated disease at diagnosis mostly underwent an intentionally less radical resection. Histopathological and molecular classification was performed according to the WHO 2021 classification [22,23]. TP53 status was determined either using immunohistochemistry (6/10 (60%) SHH-MB) or DNA sequencing (4/10 (40%) SHH-MB). Baseline and treatment-related characteristics of the patients stratified by molecular group are summarized in Table 1. The study was approved by the Institutional Ethics Committee (EK 1476/2021) and complies with the principles of the Declaration of Helsinki.

### 2.2. Intraoperative Definition of the STO

The assessment of the epicenter of the assumed origin of tumor growth was conducted based on operative reports and surgical videos by a pediatric neurosurgeon with 15 years of experience (C.D.) in accordance with the operating neurosurgeon (T.C.). Over the entire study period, the surgical strategy at our institution has been to carefully dissect the tumor as much as is feasible and obtain a clear anatomic understanding of the tumor margins, adherent neurovascular structures, growth pattern and eventually the STO before and during the actual resection. Also, these anatomical features had been systematically documented in the operative reports of all cases in this study. These reports also include clinical and radiological information. 

If the tumor was growing beneath the level of the fourth ventricular floor with subependymal extension, the STO was attributed to the brainstem, except for patients with an unequivocal tumor growth within the cerebellar nodulus-uvula.

### 2.3. Statistical Analysis

Continuous variables are reported as median and range and categorial variables as counts and percentages. Chi-square tests and Fisher’s exact tests were performed to test for association between clinicopathological features and molecular groups. Wilcoxon rank sum test and Kruskal-Wallis test were used to compare subgroups on continuous variables. Sensitivities as well as positive predictive values and 95% Wilson confidence intervals of the intraoperatively observed STO were calculated separately for each subgroup. Fisher’s exact test was used to compare the sensitivities and positive predictive values between the three molecular groups. All analyses were performed in the R Statistical Environment (Version 4.2.0.) [24]. *p* values on two-sided tests < 0.05 were considered statistically significant. 

## 3. Results

### 3.1. Intraoperative Assessment

**Figure 1:**
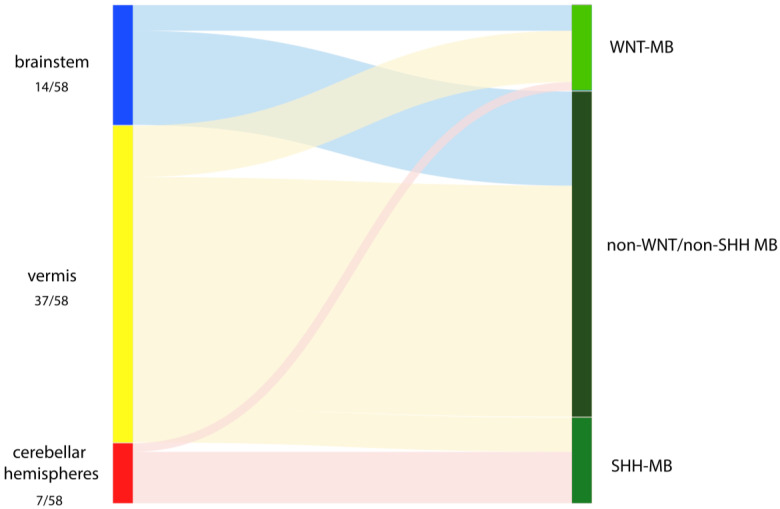
Distribution of intraoperatively assessed sites of origin dependent on their allocation to the molecular groups WNT, SHH and non-WNT/non-SHH.

The distribution of the intraoperatively inferred STOs with regard to their respective molecular groups is visualized in Figure 1 and reported in detail in Table 2.

According to the intraoperative assessment, 37/58 (64%) tumors were unequivocally assigned to originate from the vermis, with 33/37 (89%) of them originating from the lower vermis. Seven of fifty-eight tumors (12%) had a hemispheric growth.

Fourteen of fifty-eight patients (24%) had a tumor that was assigned to originate from the brainstem, with eight tumors seeming to originate either exclusively from a lateral recess or involving it, besides including the caudal rhomboid fossa in the tumor bed.

### 3.2. Relationship of Intraoperative Findings with Molecular MB Groups

**Figure 2:**
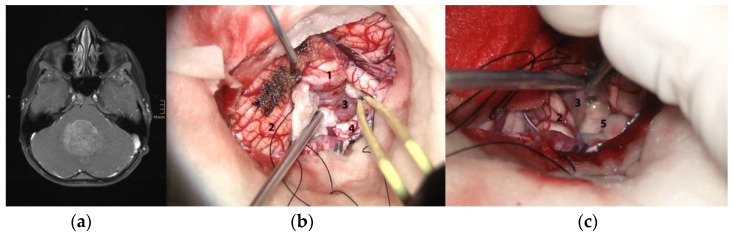
Illustrative case of a WNT-MB with vermian origin defined through intraoperative inspection: (**a**) Axial T1-contrast-enhanced MRI depicting the midline location of the tumor. (**b**) Intraoperative overview of the vermis (1), cerebellar hemispheres (2), tumor (3) and brainstem (4). The MB is replacing the nodulus of the vermis with vermian pial vessels continuing over the tumor surface. (**c**) Lifting up this tumor with a dissector clearly demonstrates that the MB is not infiltrating the floor of the fourth ventricle (5), let alone originating from it.

The intraoperative observation revealed that the majority of WNT-MB (6/10 (60%)) originated from the cerebellar lower vermis with no infiltration or even adhesion of the tumor to the brainstem (Figure 2). The location of the tumor origin of three (30%) WNT-MB could be traced to the lateral recess of the fourth ventricle, with two tumors also involving the caudal paramedian rhomboid fossa. One (10%) WNT-MB was situated entirely within the cerebellar hemisphere; the origin was classified as hemispheric.

Six (60%) SHH-MB originated from the cerebellar hemispheres, whereas four out of ten (40%) arose from the vermis. For two SHH-MB, the STO was clearly delineated in the upper vermis, from which they further extended into the hemisphere.

Of 38 non-WNT/non-SHH MB, 27 (71%) originated from the lower vermis. The origin of the remaining 11/38 tumors (29%) was assigned to the brainstem. In 7/11 (64%) of these tumors originating from the brainstem, the origin involved a unilateral recess with a slight tendency for the left side (4/7 (57%)). In 5/11 tumors (45%) arising from the brainstem, the STO showed clear infiltrative attachment to the caudal rhomboid fossa.

We further sought to identify differences in the baseline characteristics of patients with different STOs within the molecular groups. Notably, SHH-activated patients with a vermian STO were younger (median age at diagnosis (range): 1 (0–3) years) than patients with a hemispheric MB (median age at diagnosis (range): 8 (1–17) years); however, this difference did not reach statistical significance (*p* = 0.055).

### 3.3. Prediction of MB Group Based on STO

Based on the hypothesis that WNT-MB arise from the LRL, SHH-MB from the cerebellar hemisphere and non-WNT/non-SHH MB from the inferior cerebellar vermis, prediction of molecular groups based on the intraoperatively observed STO was correct in 20% for WNT-MB, 60% for SHH-MB and 71% for non-WNT/non-SHH-activated MB. The positive predictive values of the neurosurgical inspection to detect the molecular group depending on the STO were 0.21 (95% CI 0.08–0.48) for WNT-MB, 0.86 (95% CI 0.49–0.97) for SHH-MB and 0.73 (95% CI 0.57–0.85) for non-WNT/non-SHH MB. Thus, the positive predictive values of the intraoperatively observed STO differed significantly across the molecular groups (*p* = 0.002). The probability for the correct prediction of a WNT-MB based on the intraoperative observation of a tumor originating from the dorsal brainstem was significantly lower compared to SHH-MB (*p* = 0.016) and non-WNT/non-SHH MB (*p* = 0.001).

## 4. Discussion

In contrast to previous retrospective neuroradiological studies that indirectly correlated tumor location to the molecular group of MB and addressed only vague data on surgical inspection [24,25,26,27], the present study is the first that systematically assessed detailed intraoperative observations regarding the site of origin and correlated those to the molecular group in a selected group of MB patients.

The cell of origin of MB has been a matter of debate ever since the characterization of this tumor entity a century ago [28,29,30]. Molecular analyses of tumors correlated to developmental gene expression led to the concept of a group-specific origin [4]. This concept, however, has widely excluded the neurosurgical perspective, which can add valuable information through direct visual inspection [4,5,10,14,15,24,25,31,32,33]. The detailed anatomical data of the origin of MB groups provided herein from a surgical perspective are a major strength of our study. They complement the current understanding and, to some extent, seem to challenge the current concept of the molecular-group-specific cells of origin, specifically for WNT-MB. Importantly, our study lends support to the results of previous studies, which concluded that lateral location in the cerebellopontine angle was not exclusive to WNT-MB and only present in a small number of these tumors [14]. In the neuroradiological review of Patay et al., 8/16 (50%) WNT-MB showed a paramedian midline growth [10]. Based on this observation but lacking any intraoperative information that could possibly support their hypothesis, they speculated that these tumors do not originate from the vermis but the ventricular lateral recess, thereby corroborating the current developmental cell lineage concept [10]. Challenging this suggestion, the direct intraoperative inspection in our study clearly revealed that 60% of WNT-MB arose from the vermis or the cerebellar hemisphere. The ambiguous neuroradiological results given in the literature lend some support to this notion [11].

### 4.1. Intraoperatively Observed Intra-Group Heterogeneity of STO

#### 4.1.1. SHH-MB

Our study confirmed the exclusive STO of SHH-MB in the cerebellum, in accordance with this structure being a derivative of the upper rhombic lip. In contrast to the hypothesis that SHH-MB are derived from granule cell precursors predominantly found in the cerebellar hemispheres [26,33], we provide further evidence that 40% of SHH-MB originate from the vermis. Teo et al. were some of the first to critically address the concept of distinct cells of origin [24]. Although they concluded that midline tumors were significantly associated with WNT-MB and non-WNT/non-SHH MB, as suggested by Gibson et al. [4], they also showed that 47% of SHH-MB were located in the vermis, which was in contrast to the concept that SHH-MB are exclusively hemispheric [24]. Several other studies also reported that only two-thirds of SHH-MB showed a lateral hemispheric location on MRI [13,16,34]. Moreover, Grammel et al. noted that 48% of SHH-MB arose from the vermis in their neuroradiological analysis [27]. As reported in previous studies [16,25], patients with vermian SHH-MB in our study cohort were more frequently infants, which may be due to variable genetic mutations among different age groups [35,36]. Notably, two of four vermian SHH-MB were located in the upper part of the vermis, as was similarly reported previously by Wefers et al. and Dasgupta et al. [13,16]. In our cohort, the intraoperative observation of a hemispherically located tumor was predictive of the SHH-groups in 85% of the cases. As all the patients in our study were TP53-wildtype SHH-MB, potential correlations between the site of origin and the TP53 status of SHH-MB may be an interesting aspect for future research.

#### 4.1.2. WNT-MB

In only 3/10 of these tumors could the STO be intraoperatively assigned to the brainstem, while 6/10 were assessed to originate unequivocally from the lower vermis. This observation that WNT-MB preferentially grew in the midline stands in contrast to Patay et al., who reported a significant proportion of WNT-MB to be characterized by a lateralized midline position in the region of the Foramen of Luschka, and Perrault et al., who noted that 75% were located in the cerebellopontine angle cistern [10,11]. Consistent with other neuroradiological studies, our findings corroborate the hypothesis of Gibson et al. that all MB originating from the LRL are predominantly located in the midline position [34]. Similarly, Mata-Mbemba et al. found that lateralization to the cerebellopontine angle was not specific to WNT-MB, as only 3/25 unilateral cerebellopontine angle tumors were WNT MB, with group 3 and group 4 tumors accounting for the remaining 22 cases [14]. Lastowska et al. analyzed the tumor origin and location based on MR imaging and surgical reports and concluded that five of six (83%) WNT-MB had a midline location, while the remaining one was located in the cerebellopontine angle [26]. Furthermore, a recent multicenter radiogenomics study by Zhang et al. did not use lateral location as a discriminator for WNT-MB [17]. Our results further confirm the rare occurrence of a hemispheric WNT-MB. In the neuroradiological analysis of 75 WNT-MB included in the HIT trial, Stock et al. also reported 5/75 cases of WNT-MB (6.6%) with cerebellar location, which is a considerably lower proportion than that found in the present study [37].

#### 4.1.3. Non-WNT/Non-SHH MB

While the majority of these tumors grew within the lower vermis, in 7/38 cases, the tumor originated from the brainstem in the lateral recess region. The current literature suggests that non-WNT/non-SHH MB originate from precursors located in the upper rhombic lip [18,38,39]. As several Tp53 mutant murine models resembling Group 3 MB developed from cerebellar neuronal stem cells, these cells were also proposed to be the cell of origin of these groups [40]. On the contrary, in an imaging study that lacked surgical information, Wefers et al. reported that a majority of non-WNT/non-SHH MB expanded into the fourth ventricle and had contact to the cochlear and cuneate nucleus without any evidence of intracerebellar growth [13]. Consistent with these results, Narayan et al. noted that 62% of non-WNT/non-SHH MB tumors were located in the midline and had a tendency to contact the brainstem [34], which supports to some extent our observation that almost a third of non-WNT/non-SHH tumors intraoperatively arose from the brainstem. Although our findings demonstrate that a tumor originating from the vermis is highly predictive (73%) of non-WNT/non-SHH MB, vermian MB may also belong to the WNT-MB or SHH-MB.

#### 4.1.4. Clinical and Surgical Implications

The findings of our study provide evidence of an insufficient predictive accuracy of the neurosurgical perspective to correctly detect the molecular group based on the presumed tumor origin: Only 21% of all tumors that had been intraoperatively observed to originate from the brainstem were WNT-MB according to the molecular analysis. The remaining 79% of tumors arising from the brainstem were non-WNT/non-SHH MB, which are generally associated with poorer overall and progression-free survival [41]. Therefore, we conclude that, for now, maximal safe gross total resection should remain the aim of surgery irrespective of the observed STO. In compliance with our experience, it does not seem possible to reliably identify a WNT-MB intraoperatively based on a brainstem STO as developmental gene expression data would suggest. Consequently, despite the favorable progression-free and overall survival rates of WNT-MB, subtotal resection in favor of a good neurological outcome is not justified based on neurosurgical assessments. To enable a correct diagnosis intraoperatively and define the appropriate neurosurgical strategy, it will be of utmost importance to advance the development of robust and sensitive methods of intraoperative genetic methylation classification [42]. Advanced imaging techniques combined with a radio-omics approach have recently been leveraged to enable more reliable and cost-effective molecular group prediction. Using a radio-omics approach, Dasgupta et al. identified the relation of the tumor to the brainstem as a discriminating feature to predict the molecular group [16]. While the mean AUC for predicting WNT tumors using T2-weighted images was acceptable (0.75 in the training cohort and 0.69 in the validation cohort), the nomogram reached a predictive accuracy of only 41%, which was much lower compared to all other predicted molecular groups with values ranging between 56% and 95% [16]. In addition, there were large institutional differences in model performance [16]. As we demonstrated a limited correlation between WNT-MB and brainstem origin, more accurate and detailed information on the STO derived from intraoperative inspection may inform feature selection and improve the performance and accuracy of predictive models. In the most comprehensive study to date, Zhang et al. combined multiple radio-omic models in a cohort of 263 patients and achieved a high performance across all molecular groups with a micro-averaged F1 score of 88% and a binary F1 score of 98% specifically for the WNT group, thus providing a promising tool for risk-stratified clinical decision-making, which may also guide surgical decision-making preoperatively [17]. Future studies may incorporate the intraoperatively observed STO and infiltration pattern as an additional feature for the development of prognostic models based on a combination of clinical characteristics, neuroimaging, biomarkers and genetic classification data.

#### 4.1.5. Limitations

The main limitation of the study is due to the inherent nature of retrospective analyses. While we only included patients with sufficient intraoperative documentation, the neurosurgical classification was based on retrospective evaluations of surgical reports and operative videos. No attempt was made to include neuroradiological data into this retrospective study extending over a long time period, with original data only available for part of the patients and evolving imaging protocols and scanning machines resulting in a qualitative heterogeneity.

## 5. Conclusions

In our series of 58 well-documented cases, the intraoperative assessment of the STO could not reliably predict the molecular group. The current evidence still does not allow for intraoperative, group-specific risk stratification that would enable tailoring the neurosurgical strategy to the prognostic and predictive profile of the patient.

## Figures and Tables

**Table 1:** Baseline and treatment-related characteristics of the study population (n = 58) and stratified by molecular group. *p* values show the results of chi-square tests and Fisher’s exact tests, which tested for association between clinicopathological features and molecular groups.

Variable	WNT (n = 10)	SHH (n = 10)	Non-WNT/Non-SHH (n = 38)	*p* Value
Sex, n (%)				0.531
Female	5 (50.0)	4 (40.0)	12 (31.6)	
Male	5 (50.0)	6 (60.0)	26 (68.4)	
Age at diagnosis, in years, median (range)	8 (5–17)	3 (0–17)	7 (1–16)	0.128
KPS at diagnosis, median (range)	70 (20–80)	70 (60–80)	70 (60–90)	0.113
Metastases at diagnosis, n (%)				**<0.001**
M0	10 (100.0)	9 (90.0)	15 (39.5)	
M+	0 (0.0)	1 (10.0)	23 (60.5)	
Histopathological subgroup, n (%)				**<0.001**
Classic	9 (90.0)	0 (0.0)	34 (89.5)	
LCA	1 (10.0)	0 (0.0)	4 (10.5)	
DNMB	0 (0.0)	10 (100.0)	0 (0.0)	
MBEN	0 (0.0)	0 (0.0)	0 (0.0)	
TP53 status	NA	10 (100.0)	NA	NA
wildtype mutant	NA	(0.0)	NA	
Extent of resection, n (%)				**0.015**
GTR	10 (100.0)	10 (100.0)	26 (68.4)	
STR	0 (0.0)	0 (0.0)	12 (31.6)	
Radiotherapy, n (%)				**0.014**
no	0 (0.0)	5 (50.0)	6 (15.8)	
yes	10 (100.0)	5 (50.0)	32 (84.2)	
Chemotherapy, n (%)				NA
no	0 (0.0)	0 (0.0)	0 (0.0)	
yes	10 (100.0)	10 (100.0)	38 (100.0)	
KPS at last FU, median (range)	90 (60–100)	90 (20–100)	80 (30–100)	0.077
Residual disease at last FU, n (%)				0.578 1.000
Cerebral disease	1 (10.0)	2 (20.0)	3 (8.1)	
Metastatic disease	1 (10.0)	2 (20.0)	6 (16.2)	
Progression, n (%)				0.325
no	9 (90.0)	8 (80.0)	25 (65.8)	
yes	1 (10.0)	2 (20.0)	13 (34.2)	
Death, n (%)				0.881
no	9 (90.0)	8 (80.0)	34 (84.2)	
yes	1 (10.0)	2 (20.0)	6 (15.8)	

Bold type indicates statistical significance.

**Table 2:** Primary locations and extensions of the intraoperatively observed STO stratified by molecular group (n = 58). *p* values show the results of chi-square tests and Fisher’s exact tests, which tested for association between STO (brainstem, cerebellar hemispheres, and cerebellar vermis) and molecular groups.

Primary Location	Extension	WNT (n = 10)	SHH (n = 10)	Non-WNT/Non-SHH (n = 38)	*p* Value
**Brainstem**, n (%)		3 (30.0)	0 (0.0)	11 (28.9)	0.196
Caudal rhomboid Fossa, n					
	*including*				
	left lateral recess	1	0	0	
	right lateral recess	0	0	2	
	bilateral lateral recesses	1	0	3	
Lateral recess, n					
	*including*				
	left lateral recess	1	0	3	
	left lateral recess and cerebellar peduncle	0	0	1	
	right lateral recess	0	0	1	
Cerebellar peduncle, n (%)		0	0	1	
**Cerebellar hemispheres**, n (%)		1 (10.0)	6 (60.0)	0 (0.0)	**<0.001**
**Cerebellar vermis**, n (%)		6 (60.0)	4 (40.0)	27 (71.1)	0.226

Bold type indicates statistical significance.

## Data Availability

The data presented in this study are available on request from the corresponding author.
